# Parents’ use of coercive and indulgent feeding practices for children with avid eating behaviour: an Ecological Momentary Assessment study

**DOI:** 10.1186/s12966-025-01715-w

**Published:** 2025-02-07

**Authors:** Abigail Pickard, Katie L. Edwards, Claire Farrow, Emma Haycraft, Moritz Herle, Clare Llewellyn, Helen Croker, Alice Kininmonth, Jacqueline Blissett

**Affiliations:** 1 School of Psychology, Institute of Health and Neurodevelopment, Aston University, Birmingham, UK; 2 Department of Clinical Psychology, School of Health in Social Science, University of Edinburgh, Edinburgh, EH8 9AG UK; 3 School of Psychology, University of Birmingham, Birmingham, UK; 4 School of Sport, Exercise and Health Sciences, Loughborough University, Loughborough, UK; 5 Social, Genetic & Developmental Psychiatry Centre, Institute of Psychiatry, Psychology & Neuroscience, King’s College London, London, UK; 6 Research Department of Behavioural Science and Health, Institute of Epidemiology and Health Care, University College London, London, UK; 7 World Cancer Research Fund International, London, UK; 8 School of Food Science and Nutrition, Faculty of Environment, University of Leeds, Leeds, UK; 9 Leeds Institute for Data Analytics (LIDA), Faculty of Medicine and Health, University of Leeds, Leeds, UK

**Keywords:** Ecological Momentary Assessment (EMA), Avid eating, Preschoolers, Eating behaviour, Coercive/indulgent feeding

## Abstract

**Background:**

Children with avid eating behaviour display high food responsiveness, high emotional overeating and low sensitivity to fullness; behaviours which may increase the risk of obesity and are challenging for parents to manage. This study explores the situational predictors of coercive or indulgent feeding practices among parents of children with avid eating behaviours using Ecological Momentary Assessment (EMA).

**Methods:**

The study involved 109 parents of 3-5-year-old children exhibiting avid eating behaviour. Over 10 days, participants completed EMA surveys via a mobile app to report on their mood, stress, feeding goals, and feeding practices during eating occasions. Multilevel modelling was used to assess how parental mood, goals, and the eating context (e.g., meal versus snack, public versus private setting) influenced feeding practices.

**Results:**

Parents were more likely to use specific coercive or indulgent feeding practices when experiencing higher stress, when aiming to avoid mealtime conflict, and during meals versus snacks. A negative meal atmosphere and a public setting also increased the likelihood of certain indulgent practices. Notably, parents were more likely to report giving their child food to calm them down or help manage their behaviour when the meal atmosphere was perceived as negative and if they aimed to reduce conflict at the meal. The findings highlight that the context of feeding occasions significantly drives the use of coercive or indulgent feeding practices.

**Conclusions:**

Parental stress, goals, and the eating context are key determinants of coercive or indulgent feeding practices with children exhibiting avid eating behaviours. Interventions to support parents should consider these dynamic factors, promoting healthier feeding strategies tailored to real-life contexts.

**Supplementary Information:**

The online version contains supplementary material available at 10.1186/s12966-025-01715-w.

## Introduction

Early childhood is a critical period for establishing long-term healthy eating behaviours. However, children’s appetitive traits, which are both environmentally and genetically determined, impact both the quantity and quality of food consumed [1–4]. Approximately 20 per cent of pre-schoolers living in the UK display ‘avid eating’ behaviours [5], marked by a higher enjoyment of food, increased responsiveness to food cues, a tendency to overeat in response to emotions, quicker eating, lower sensitivity to fullness, and lower food fussiness in comparison to other children. Longitudinal studies have shown a positive correlation between such eating behaviours and subsequent adiposity, indicating that these children might be at higher risk of obesity [3]. Parents of children with avid eating behaviour report frequent food demands from their children and that the management of this can be challenging [6]. To manage their child’s increased interest and responsiveness to food, parents may employ specific feeding practices (FPs).

Our work has demonstrated that parents of children with avid eating behaviours report using more coercive FPs, such as restricting food and using food to regulate emotions, than parents of children with less avid eating behaviours [5]. A parent might engage in food restriction (i.e., limiting the types or amounts of foods their child can eat) to help them avoid overconsumption of certain foods [7]. Longitudinal evidence has also demonstrated that coercive or indulgent FPs, such as pressure to eat and providing food to soothe a child’s emotions, are used *in response to* increased appetite avidity (such as high food responsiveness, enjoyment of food, and emotional overeating) in young children, suggesting that this relationship is likely bi-directional [8]. Further investigation also demonstrates that parents’ use of coercive or indulgent FPs can result in greater appetite avidity and food approach tendencies in children in later years [9]. Additionally, both coercive and indulgent practices have been associated with less healthful dietary intake (e.g., sugar-sweetened beverages), a higher body mass index, and the development of maladaptive eating behaviours, such as emotional eating or binge eating [10, 11]. Therefore, understanding the predictors of the use of both coercive and indulgent practices is important to enable the development of tailored and effective intervention strategies.

Most research on parental FPs relies on static, self-reported measures that do not capture the variability in parental behaviour over time and in different contexts. However, research has shown that parental FPs vary by time and context, highlighting the complexity of parent-child feeding dynamics [7, 12]. Although momentary FPs have not yet been examined in children with avid appetites, qualitative research suggests that such practices vary depending on the situation, such as the type and location of eating [13]. For example, parents demonstrate less control over the timing, as well as the types and amounts of food eaten during snacks, compared with the control exhibited during meals [14]. A qualitative study conducted with parents of children displaying avid eating suggested that parents adopt different FPs in response to contextual factors, such as time of day and special occasions, and use more controlling FPs during snack times compared to mealtimes [6].

Thus, examining the predictors of the use of coercive or indulgent FPs ‘in-the-moment’ is warranted to allow a more precise examination of time and context effects on feeding interactions as they happen. Ecological Momentary Assessment (EMA) is becoming a popular research method to investigate momentary feeding interactions between parents and children as they occur in their natural environment (e.g [7, 12, 15, 16]. Unlike traditional assessments that rely on retrospective reporting, EMA involves participants responding to surveys at various random or scheduled times throughout the day, over several days or weeks. This method aims to capture authentic, moment-to-moment variations and provides a more accurate and detailed picture of daily life experiences [17, 18].

EMA approaches have recently been used to examine the contextual predictors of momentary FPs in children, but without accounting for the child’s appetite avidity or eating behaviour [7, 12]. This research has shown that fluctuations in parental mood and stress throughout the day affect subsequent FPs [7]. Qualitative research also indicates that parents employ specific feeding strategies to manage challenging interactions with children, such as using indulgent strategies when their energy is low [19]. For example, higher maternal stress and depressed mood earlier in the day can predict increased pressure to eat and less likelihood of serving homemade meals in the evening [20].

In addition to context and parental emotion, parental cognitions, such as parental feeding goals, also play a role in feeding interactions [21]. Parents of 6–10-year-old children with fussy eating often have feeding goals beyond health, such as avoiding mealtime stress, conflict and hunger, limiting unhealthy foods, and involving their children in food preparation [22]. However, the main feeding goals associated with coercive or indulgent FPs in children with avid eating behaviour have yet to be established. For example, parents may use greater restriction of unhealthy foods when health-related goals are prioritised or use indulgent FPs, such as catering to the child’s preferences, when conflict avoidance is their main goal. The prioritised feeding goal of a parent at a specific eating occasion is also likely to be influenced by the parent’s mood and the eating context, such as aiming to reduce conflict when in public or in the presence of other people.

In summary, while the existing literature gives useful insight into parents’ use of FPs within specific contexts, at present no research has investigated momentary feeding interactions between parents and children with avid eating behaviour, who express greater food demands.

The hypotheses that this study tested were as follows:

H1: Parents who report higher levels of stress or negative affect at one measurement occasion will be more likely to report using coercive or indulgent FPs at the subsequent feeding occasion.

H2: Parents who report feeding goals of reducing mealtime conflict will be more likely to report using coercive or indulgent FPs.

H3: Parents are more likely to report using coercive or indulgent FPs for meals rather than snacks, when in public and in the presence of others versus when at home, and when they report the atmosphere as tense/stressful.

## Methods

This EMA study was part of the broader APPETItE project, which investigates feeding and eating behaviours in 3-5-year-old children with avid eating behaviour. Recruitment, enrolment, and data collection were completed from October 2023 to April 2024. The study hypotheses, design and analysis plan were pre-registered before data collection (see 10.17605/OSF.IO/N48YV).

### Participants

Due to the novelty of this research, a precise power calculation was not feasible. Therefore, based on previous EMA research, we aimed to invite 200 parents to ensure sufficient data for examining within- and between-subject effects while accounting for attrition [23]. Parents were recruited via the online participant panel Prolific (for complete details on the recruitment procedure please see the published study protocol [24]). Parents of children aged 3–5 years completed the Children’s Eating Behaviour Questionnaire (CEBQ; [25]) to determine whether their child displayed an avid eating profile using Latent Profile Analysis. Based on the latent profiling 313 parents had a child assigned to the avid eating profile and were invited to take part in the study. Eligibility criteria necessitated that, in addition to having a child with an avid eating behaviour profile, the parents had a good understanding of English, lived in the United Kingdom and were responsible for feeding their child more than half the time when at home. Parents of children who were autistic, had severe learning disabilities, or a chronic illness that directly affected their dietary needs and eating habits were not eligible to participate. 147 participants completed the initial survey to register for the EMA survey period. One participant indicated that their child was above the age range for the study (82 months old), so they were excluded from all further data analysis. Parents re-reported their child’s eating behaviour using the CEBQ just before commencing the EMA period. Of the 146 participants, 109 children (74.7%) remained assigned to the ‘avid eating’ profile, 31 (21.2%) were now assigned to the ‘typical eating’ profile and 6 (4.1%) were now assigned to the ‘happy eating’ profile. Only the children assigned to the ‘avid eating’ profile were retained in the following data analysis, per the preregistration.

### Procedure

Participation in this study was remote; surveys were administered through a mobile smartphone app downloaded directly to parents’ smartphones. Parents were informed that if they did not have a compatible smartphone, they could request one from the research team to use for the study period. Parents completed a baseline questionnaire, 10 days of EMA, and an end-of-study questionnaire. Parents received a £100 (approximately US $126) shopping voucher if at least 8 days were complete, the reimbursement was pro-rated for each complete day.

#### Baseline

The baseline questionnaire gathered information about parent and child characteristics (e.g., demographics, socioeconomic class, food insecurity) and general parent mood and wellbeing. The findings are reported elsewhere (Pickard et al., under review).

#### EMA period

Parents completed a 10-day EMA period, comprised of both signal contingent (researcher-initiated surveys) and event contingent surveys (participant-initiated surveys), to examine their mood, emotions, and feeding experiences as they happen (see Table 1 for example measures).

#### Signal-contingent surveys

Participants were notified of the signal-contingent surveys through push notifications scheduled to the participant’s smartphone. Parents received four signal-contingent surveys each day at semi-random times within four specific 120-minute windows: 7–9 AM (morning survey), 10 AM–12 PM, 1–3 PM, and 4–6 PM. From the first notification, parents had 60 min to complete the surveys before the link expired. The signal-contingent surveys assessed the parents’ current mood, level of stress, and context of what they were doing and who they were with. Items measuring positive affect (e.g., I feel happy), negative affect (e.g., I feel sad), and stress (e.g., I feel tense) were measured on a five-point Likert scale with 1= ‘not at all’, 2 = ‘a little’, 3 = ‘moderately’, 4 = ‘quite a bit’, 5 = ‘extremely’.

#### Event-contingent surveys

Event-contingent (food) surveys were self-initiated by parents each time their child asked for or consumed food while the parent was present (see Table 1). The food surveys examined parental FPs used during the eating occasion, adapted from the Real-Time Parent FPs measurement tool [23]. The individual FPs were treated as binary outcome variables with 1 = present and 0 = absent (see Table 1).

**Table 1:** Coercive and indulgent feeding practice (outcome variables)

**Coercive FPs**	
PFP1: Encourage child to eat more food than they wanted to (pressure to eat).	
PFP2: Offer child food as a reward for eating more (threats & bribes).	
PFP3: Have to make sure your child did not eat too much food (restriction).	
PFP4: Offer child a treat or reward for trying a new food (threats & bribes)	
PFP5: Trick or bribe child into eating more than they wanted to (threats & bribes).	
**Indulgent FPs**	
PFP6: Prepare separate food that you knew child would enjoy (anticipatory catering).	
PFP7: Allow child to choose a separate meal or different food because they did not want to eat what was offered (unstructured practices)	
PFP8: Give child food to calm them down or help manage their behaviour (using food to control negative emotions).	

Table 2 outlines the predictor variables including the feeding goal of the parent, adapted from the Family Mealtime Goals Questionnaire [21], and the context of the meal adapted from the EMA component of the Family Matters study [16].

**Table 2:** Predictor variables and coding Scheme

	Response options	Dummy code
**Feeding Goal**		
To avoid arguments about food at mealtimes	1 = Yes	Yes
	0 = No	**No**
**Meal Context**		
Meal child was eating	1 = Breakfast	**Meal**
	2 = Lunch	**Meal**
	3 = Evening meal	**Meal**
	4 = Snack	Snack
Where eating event took place	1 = Around a table at home	**Private**
	2 = On the sofa/chair in living area	**Private**
	3 = Scattered throughout the house	**Private**
	4 = In the car	**Private**
	5 = At a restaurant	Public
Atmosphere of eating event	1 = Chaotic	Negative
	2 = Rushed	Negative
	3 = Tense	Negative
	4 = Relaxed	**Positive**
	5 = Enjoyable	**Positive**
	6 = Neutral	Neutral

NB. Bolded items indicate reference variables The reference variables were decided based on the factors we believed would not be associated with the use of coercive/indulgent FPs

### Data analyses

Descriptive statistics of demographic variables were performed to characterise the sample. To test the main hypothesis, data were analysed using multi-level modelling in R version 4.4.1 with the package ‘lme4’ [26]. As per the pre-registration, only the coercive or indulgent FPs were included as outcome variables in this analysis. For each model, we analysed only eating occasion observations where the parent had completed a signal contingent survey within four hours before the eating occasion. Parent stress and negative affect were centred for each participant to improve the interpretation of the findings. To allow for sufficient sample sizes within the mixed models the categorical items included in the model were re-coded to binary variables (see Table 1). P-values were used to determine whether models were significantly different from zero (*p* <.05). The models included a random intercept that was allowed to vary within individuals and a random slope that was allowed to vary within individuals. For the parent’s mood, we centred each predictor variable around a person’s mean allowing us to observe trait and state effects. The state components of negative affect, positive affect and stress were centred for each participant (mean of 0), where positive deviations (e.g., 1.2) are greater than the participant’s average levels, and negative deviations (e.g., -0.8) are less than their usual levels. The outcome variables (5 x coercive and 3 x indulgent FPs) were run in separate models to allow a nuanced understanding of what factors influenced specific FPs.

## Results

### Demographics

The 109 parents had a mean age of 34.6 years (SD = 5.5, min = 24.8, max = 55.3). Children had a mean age of 53.1 months (SD = 10.3, min = 36.8, max = 71.43). Mean scores for participant-centred positive affect, negative affect, and mood were 2.74 (SD = 0.61), 1.77 (SD = 0.6), and 2.24 (SD = 0.64), respectively (a maximum score of 5 indicates the greatest level of endorsement for these three constructs). An overview of the parents’ demographic background and child sex is provided in Table 3.

**Table 3:** Demographic details of the sample (*N* = 109)

	*N*	Per cent
**Child Sex**		
Male	50	45.9
Female	59	54.1
**Parent Sex**		
Male	24	22
Female	85	78
**Parent Ethnicity**		
Asian	8	7.3
Black	7	6.4
White	91	83.5
Mixed	1	1.0
Other	2	1.8
**Education**		
Degree	64	58.7
No-degree	45	41.3
**Working Status**		
Unemployed	21	19.3
Working part-time (between 8–29 h per week)	35	32.1
Working full-time (30 h or more per week)	53	48.6
**Adequacy of Income**		
Living Comfortably	47	43.1
Managing	46	42.2
Finding it Difficult	9	8.3
Finding it Very Difficult	6	5.5
**Household Food Security**		
High or Marginal Food Security	72	66.1
Low Food Security	16	14.7
Very Low Food Security	21	19.3

### Eating occasions

Parents reported on 1777 in-the-moment eating occasions in which their child ate food, Table 4 displays the overall frequencies of reported use for each feeding practice. Meals accounted for 1271 (71.5%) and snacks accounted for 506 (28.5%) of eating occasions. Approximately one-third of eating occasions (595, 33.5%) were initiated by the child requesting or helping themselves to food. The majority of meals occurred in the home (1659, 97%) and in the presence of other family members (1259, 70.8%). Parents reported that 73.4% (1305) of the eating occasions had a positive atmosphere, 12.9% (230) had a neutral atmosphere and 13.6% (242) had a negative atmosphere.

**Table 4:** Reported use of feeding practices for 1777 eating occasions

Feeding practice	Reported use	Percentage of use for 1777 eating occasions
PFP1: Pressure to eat	323	18%
PFP2: Food reward for eating	119	7%
PFP3: Restriction	212	12%
PFP4: Food reward for new food	111	6%
PFP5: Trick/bribe	116	7%
PFP6: Prepare preferred food for child	363	20%
PFP7: Child to choose different food	142	8%
PFP8: Food to soothe	143	8%

**Fig. 1:**
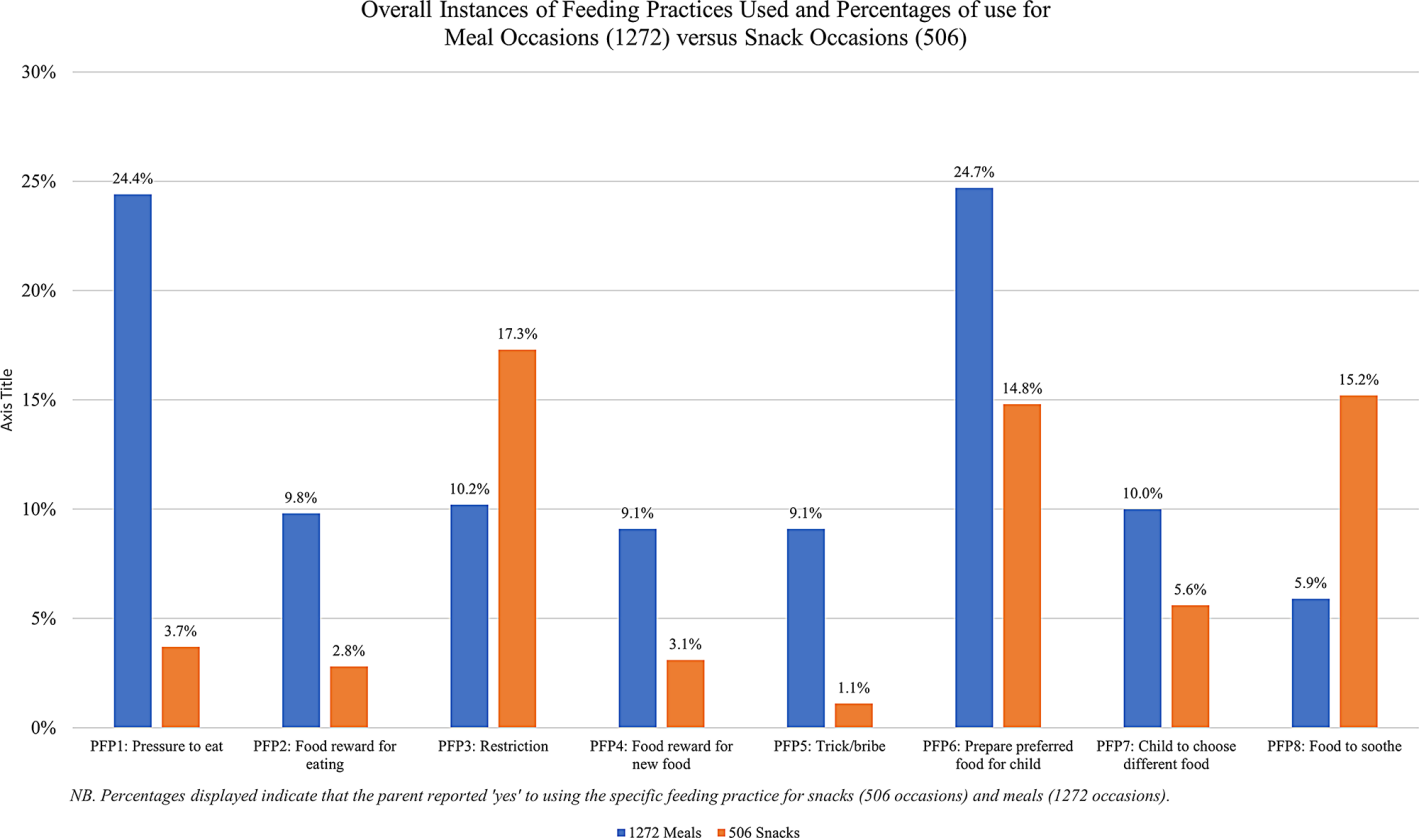
Overall frequencies of FPs used (Ns) and percentages of use for meals versus snacks

Figure 1 displays the number of instances parents reported each feeding practice out of the 1777 eating occasions. The most commonly reported feeding practice was indulgent: preparing separate food that the parent knew the child would enjoy (363 instances, 20.4% of eating occasions) while the least common was coercive: offering the child a reward for trying a new food (111 instances, 6.2% of eating occasions). The percentage of coercive or indulgent practices used differed for whether the eating occasion was a meal or a snack (see Fig. 1).

### Momentary effects

To evaluate temporal ordering, data collected from EMA event prompts (i.e., participant-initiated surveys of food parenting practices used at specific eating occasions) were paired with data from EMA signal prompts (i.e., researcher-initiated surveys of parent stress and parent mood) collected up to four-hours earlier on the same day for each participant. There were 903 paired entries, with a mean elapsed time of 73.3 min (SD = 71.4 min, min = 0 min, max = 239 min) between the signal mood/stress survey and the subsequent eating event.

A parent’s level of stress before the eating event, endorsing a feeding goal of avoiding arguments over food, a negative atmosphere, meal versus snack, at home or in public, and the child’s probability of assignment to an avid eating profile were all significant predictors for at least one of the coercive/indulgent FPs (Table 5 presents the model coefficients and significance level, see Supplementary Tables S1-S8 for full details of the model coefficients).

**Table 5:** Model coefficients for each coercive parental feeding practice (PFP1-PFP5) or indulgent parental feeding practice (PFP6-PFP8)

		Fixed effects (*p*-value)							
		PFP1: Pressure to eat	PFP2: Food reward for eating more	PFP3: Restriction	PFP4: Food reward for eating new food	PFP5: Trick or bribe	PFP6: Prepare preferred food	PFP7: Child to choose different food	PFP8: Food to soothe
**Intercept**		-1.98 (0.21)	-5.85 (0.02)	-3.27 (0.1)	-3.87 (0.16)	-4.05 (0.09)	-**3.57 (0.04)**	-2.82 (0.21)	-4.51 (0.08)
**State stress**		**0.6 (0.02)**	-0.31 (0.41)	0.08 (0.81)	0.34 (0.4)	0.21 (0.58)	0.26 (0.33)	0.53 (0.2)	0.51 (0.19)
**State Negative Affect**		-0.38(0.32)	0.71 (0.22)	− 0.24 (0.61)	0.12 (0.85)	-0.43 (0.48)	-0.14 (0.72)	-0.37 (0.52)	-0.2 (0.73)
**Reducing Conflict Goal**									
Yes		0.27 (0.28)	**1.04 (0.01)**	0.53 (0.08)	0.38 (0.35)	**0.99 (0.01)**	**0.82 (< 0.001)**	**1.86 (< 0.001)**	**1.47 (< 0.001)**
No (referent)		-	-	-	-	-	-	-	-
**Atmosphere**									
Negative		**0.6 (0.05)**	0.85 (0.06)	-0.13 (0.72)	0.49 (0.32)	0.42 (0.36)	-0.32 (0.35)	-0.58 (0.29)	**1.06 (0.01)**
Neutral		0.1 (0.77)	-0.96 (0.14)	-0.08 (0.83)	-0.98 (0.12)	-0.68 (0.22)	0.03 (0.94)	-0.66 (0.26)	-0.1 (0.84)
Positive (referent)		-							
**Type of eating occasion**									
Snack		**-2.52 (< 0.001)**	**-1.94 (< 0.001)**	**0.97 (< 0.001)**	**-2.05 (< 0.001)**	**-3.14 (< 0.001)**	-0.26 (0.31)	**-0.94 (0.05)**	**1.78 (< 0.001)**
Meal (referent)		-	-	-	-	-			
**Location**									
Public setting		-0.43 (0.47)	0.07 (0.93)	-0.13 (0.86)	0.28 (0.74)	-0.64 (0.4)	-0.02 (0.98)	**2.11 (< 0.001)**	1.12 (0.14)
Private setting									
**Probability of avid eating**		0.71 (0.51)	0.5 (0.78)	0.56 (0.65)	-0.17 (0.93)	**3.23 (0.05)**	1.14 (0.31)	-1.16 (0.43)	**3.79 (0.03)**
**Day of the Week**									
Weekend		0.19 (0.42)	0.6 (0.1)	0.0 (0.99)	**0.95 (0.01)**	**0.78 (0.02)**	0.41 (0.07)	**0.87 (0.01)**	0.26 (0.47)
Weekday (referent)		-	-	-	-	-	-	-	-

NB. PFP = Parental Feeding Practice. Bold values indicate a significant effect at *p* <.05. Fixed effects were estimated from mixed-effects linear regressions with the presence of each feeding practice as the outcome. Participant ID was included as a random effect to account for parent-to-parent variability in their use of feeding practices

In answer to hypothesis 1, a higher score of stress in the four hours before the eating occasion was associated with an increased probability of encouraging their child to eat more food than they wanted to (PFP1). There were no other relationships between stress/mood and subsequent feeding practices.

Aligned with hypothesis 2, when parents reported the goal of avoiding conflict, they were more likely to report offering their child a reward for eating more food (PFP2), tricking or bribing their child into eating more than they wanted to (PFP5), prepare separate food that they knew their child would enjoy eating (PFP6), allowing their child to choose a separate meal or different food because they did not want to eat what was offered (PFP7), or giving their child food to calm them down or help manage their behaviour (PFP8).

Results of hypothesis 3 were somewhat mixed. When the eating occasion was a snack rather than a meal, parents were more likely to report having to make sure that their child did not eat too much food (PFP3) or giving their child food to calm them down or help manage their behaviour (PFP8). In the snack context, parents were less likely to pressure their child to eat more food than they wanted to (PFP1), offer their child a reward for eating more food (PFP2), offer their children a treat or reward for trying new food (PFP4), trick or bribe their child into eating more than they wanted to (PFP5), or allow their child to choose a separate meal or different food (PFP7). Allowing the child to choose a different food (PFP7) was the only feeding practice reported more when in public than in a private setting. When the eating situation was perceived as negative compared to neutral or positive, pressuring a child to eat more (PFP1) and giving a child food to calm them down or help manage their behaviour (PFP8) were reported more often. At the weekend, offering their children a treat or reward for trying new food (PFP4), tricking or bribing their child into eating more than they wanted to (PFP5), and allowing the child to choose a different food (PFP7) were more commonly reported compared to a weekday. Parents of a child with a higher probability of avid eating behaviour were more likely to give their child food to calm them down or help manage their behaviour (PFP8) and trick or bribe their child into eating more than they wanted to (PFP5).

## Discussion

The present study explored the contextual and situational factors influencing parental coercive or indulgent feeding practices (PFPs) among parents of preschool children with avid eating behaviour using ecological momentary assessment (EMA).

Hypothesis 1 was partly supported by the findings, in that higher parental stress predicted subsequent use of greater pressure to eat. These findings align with previous research suggesting that stress can deplete a parent’s emotional resources, leading them to adopt more controlling or compensatory feeding strategies [19, 20]. Conversely, parental mood was not a significant predictor of the use of any coercive or indulgent PFPs. This suggests that the momentary experience of stress, rather than mood, was a more important determinant of subsequent feeding practices in our sample. It is also possible that when asked to report stress in the moment, parents were actually reporting on a more stable or pervasive experience than mood, which may help to explain why mood ratings were less predictive of subsequent behaviour within the four-hour window between recording parents’ states and the subsequent eating event.

In support of hypothesis 2, feeding goals aimed at reducing mealtime conflict led to both indulgent (e.g., soothing with food) and coercive (e.g., food rewards) practices, emphasizing the role of non-health-related goals in shaping less adaptive strategies. Our finding supports the notion that feeding goals beyond health, such as minimising stress or conflict, play a crucial role in the use of less adaptive parental FPs [21].

As predicted in hypothesis 3, context also influenced PFPs, with effects of snack vs. meals, public vs. private, negative vs. positive mealtimes, and weekends vs. weekdays all being evident. In comparison to snacks, meals appeared to be more likely occasions for introducing new foods, given that in this context parents reported more use of coercive practices such as offering their child a treat or reward for trying new food. In contrast, snacks were often used for immediate emotional regulation due to their spontaneous and rewarding nature. Unlike meals, which usually have predefined portions and follow a structured routine, snacks can be more spontaneous and are often highly palatable, convenient and commonly designed to be rewarding and appealing (such as sweets, crisps, or cookies). Together, these features of snacks may lead parents use snacks to regulate a child’s emotions because of their effectiveness in providing immediate gratification, particularly in children with avid eating tendencies. Public settings prompted the more indulgent practice of allowing children to choose different foods to avoid conflict. Public settings may present additional pressures and social expectations, prompting parents to use less adaptive practices to manage their children’s behaviour [16]. Unsurprisingly, parental perception of negative mealtimes predicted greater use of pressure to eat as well as the indulgent practice of giving food to soothe. These findings are consistent with the literature examining the bidirectional effects of pressure to eat and more difficult feeding interactions [27] as well as the commonly observation that parents are more likely to use food to soothe when their children have more negative affect or are difficult to manage due to temperamental traits such as surgency [28]. Finally, at weekends, parents were more likely to allow the child to choose a different food to that which is offered, which might suggest a more indulgent approach to weekend eating opportunities, but weekends also shared some more coercive feeding practices in common with weekday mealtimes, designed to get children to eat new foods. In summary, our study demonstrates the powerful effects of contextual factors on feeding practice, and calls for a more nuanced understanding of why, which and when specific feeding practices are used by parents.

In addition to these external contextual effects, we also observed effects of children’s appetite on feeding practices, even within this sample chosen for their homogeneity in avid eating. Notably, parents of children with higher probability scores of avid eating behaviour were more likely to give food to calm children or manage their behaviour and to trick or bribe their child into eating more than the child wanted to. This finding underscores the reciprocal nature of parent-child feeding dynamics, where children’s avid eating behaviours may evoke more indulgent responses from parents, potentially perpetuating a cycle of maladaptive eating behaviours, such as emotional overeating. While instrumental FPs, such as using coercive methods to encourage eating and using food to soothe, may be effective in the short term, longitudinal evidence has demonstrated that such practices are associated with increased emotional overeating in later childhood [9, 29, 30], which in turn increases risk of obesity [31].

Our novel findings provide insights into the dynamic and context-dependent nature of feeding interactions, offering valuable implications for interventions aimed at parenting children with avid eating behaviour who are at greater risk of adiposity in later life. This study’s findings have several important implications for the design of interventions aimed at preventing childhood obesity and promoting healthy eating behaviours. First, interventions should address parental stress and conflict reduction as critical components. Providing parents with tools to manage their stress could reduce the reliance on coercive or indulgent FPs. Mindfulness-based stress reduction and cognitive-behavioural strategies could be beneficial in this regard [20]. Second, interventions should focus on helping parents set and adhere to consistent feeding goals or more structured FPs to prevent mealtime conflict in the first instance. Practical strategies need to be developed that explain how consistent, health-oriented FPs, even in the face of immediate challenges, could help parents resist the urge to use food as a tool for immediate emotional regulation or conflict avoidance [21]. Third, given the significant impact of meal settings and atmospheres, interventions should provide parents of children with avid eating strategies for managing feeding in various contexts. This could include practical tips for handling public eating situations and creating a positive mealtime atmosphere at home, even during busy or stressful times [16]. Finally, this research underscores that meals and snacks are very different eating events with different feeding goals and practices. As such, future interventions need to tailor feeding advice around whether it is a meal or snack.

### Limitations

While the EMA methodology allowed for the real-time capture of FPs and contextual factors, there are limitations to consider, such as the reliance on parent-reported data which may introduce social desirability bias. Due to the reliance on parent reports, we also decided not to collect anthropometric data such as the child’s BMI, which is often inaccurately reported by parents [32]. However, child BMI has been documented to have bi-directional associations with parental feeding practices and it would have been beneficial to include directly measured BMI as a potential covariate in this study [11].

Furthermore, although the proportion of ethnicity is representative of the UK [33] the participants were predominately White which may limit the generalisability of findings. Additionally, the sample may not be fully representative of all parents of children with avid eating behaviours particularly as children displaying very high appetite avidity may place a greater burden on parents which would hinder the parent from completing the surveys in-the-moment.

### Future research

This study has underlined the importance of in-the-moment data capture for understanding the complex interactions between context and feeding practices. Further work using this methodology could incorporate greater assessment of the role of the child’s eating behaviour or temperament into this type of analysis, given that these factors are commonly implicated in the prediction of parentally reported feeding practices captured with traditional questionnaire methods [5]. Longitudinal studies using a series of EMA analyses could provide further insights into how parental FPs evolve and their long-term impact on children’s eating behaviours and weight outcomes. There are also implications of our study for measurement, given that the widely used questionnaire measures of feeding practice currently fail to capture this variation by context [34]. In particular, the substantial impact of snack vs. mealtime context on feeding practices observed in this study highlights the importance of developing measures which examine feeding practices separately for those contexts.

While food security was not a focus of our study, we saw high rates of food insecurity in our sample, and this was reported in more families than reported that their income made it difficult or very difficult to manage. This was also observed in our previous study of UK families where families with a child who shows avid eating also reported higher levels of food insecurity without significantly higher levels of socio-economic deprivation [5]. This leads us to suspect that a child’s persistent requests for food and greater frequency of eating leads parents to either experience greater food insecurity or that this challenge becomes more salient in the context of repeated child requests. More research should investigate perceptions of food insecurity of parents with children with avid eating, and examine the interaction of static vs. dynamic contextual effects to predict feeding practices. Finally, exploring the factors that predict the use of more structured and autonomy support FPs is crucial to harnessing the opportunities for employing more beneficial FPs.

## Conclusion

This study underscores the complex and dynamic nature of parental FPs among caregivers of children with avid eating behaviours. The results of the Ecological Momentary Assessment demonstrate that parental stress, type of eating occasion, eating location, and feeding goals predict the use of coercive or indulgent FPs by parents of children with avid eating. By highlighting the influence of stress, mood, feeding goals, and contextual factors, our findings contribute to a more nuanced understanding of feeding dynamics and offer valuable directions for interventions for children with avid eating tendencies. Supporting parents in managing their stress, setting consistent feeding goals, and navigating various feeding contexts can play a crucial role in promoting healthier feeding behaviours and preventing poorer dietary habits and health outcomes in young children.

## Electronic supplementary material

Below is the link to the electronic supplementary material.

Supplementary Material 1

## Data Availability

To foster transparency and replicability in science, all our hypotheses and study designs were preregistered and received ethical approval prior to data collection. The data sets generated and analysed during this study will be available from the corresponding author upon reasonable request.

## Abbreviations

FPs: Feeding practices
EMA: Ecological Momentary Assessment
CEBQ: Child Eating Behaviour Questionnaire (Wardle et al., 2001)
