# Supplementary material for: Parents’ use of coercive and indulgent feeding practices for children with avid eating behaviour: an Ecological Momentary Assessment study
Source: Int J Behav Nutr Phys Act. 2025 Feb 7;22:16. doi: 10.1186/s12966-025-01715-w (PMC11803941; doi:10.1186/s12966-025-01715-w)
Supplement: Supplementary file 1 — Supplementary Material 1 [file 12966_2025_1715_MOESM1_ESM.docx]

**Supplementary material**

| **Table S1 - PFP1: Have to encourage your child to eat more food than they wanted to** | | | | |
| --- | --- | --- | --- | --- |
| Model Term | Coefficient | Std. Error | t | Sig. |
|  |  |  |  |  |
| **Intercept** | -1.984 | 1.5698 | -1.264 | .207 |
| **State stress** | .604 | .2544 | 2.373 | .018 |
| **State Negative Affect** | -.380 | .3854 | -.987 | .324 |
| **Reducing Conflict Goal =1** | .270 | .2495 | 1.083 | .279 |
| **Reducing Conflict Goal =0** | 0^b^ | . | . | . |
| **Negative atmosphere** | .596 | .2966 | 2.009 | .045 |
| **Neutral atmosphere** | .100 | .3494 | .288 | .774 |
| **Positive atmosphere** | 0^b^ | . | . | . |
| **Snack** | -2.516 | .4084 | -6.159 | <.001 |
| **Meal** | 0^b^ | . | . | . |
| **Public setting** | -.425 | .5876 | -.723 | .470 |
| **Private setting** | 0^b^ | . | . | . |
| **Probability of avid eating** | .709 | 1.0834 | .655 | .513 |
| **Parent age** | -.016 | .0316 | -.494 | .621 |
| **Child age** | .008 | .0182 | .454 | .650 |
| **Weekend** | .187 | .2322 | .806 | .420 |
| **Weekday** | 0^b^ | . | . | . |
| b. This coefficient is set to zero because it is redundant | | | | |

| **Table S2. PFP2: Offer your child food as a reward for eating more** | | | | |
| --- | --- | --- | --- | --- |
| Model Term | Coefficient | Std. Error | t | Sig. |
|  |  |  |  |  |
| **Intercept** | -5.854 | 2.5701 | -2.278 | .023 |
| **State stress** | -.313 | .3783 | -.828 | .408 |
| **State Negative Affect** | .709 | .5780 | 1.228 | .220 |
| **Reducing Conflict Goal =1** | 1.041 | .4142 | 2.513 | .012 |
| **Reducing Conflict Goal =0** | 0b | . | . | . |
| **Negative atmosphere** | .848 | .4563 | 1.858 | .064 |
| **Neutral atmosphere** | -.962 | .6537 | -1.472 | .141 |
| **Positive atmosphere** | 0b | . | . | . |
| **Snack** | -1.942 | .5549 | -3.501 | <.001 |
| **Meal** | 0b | . | . | . |
| **Public setting** | .071 | .8471 | .084 | .933 |
| **Private setting** | 0b | . | . | . |
| **Probability of avid eating** | .496 | 1.8066 | .275 | .784 |
| **Parent age** | -.037 | .0489 | -.755 | .450 |
| **Child age** | .062 | .0286 | 2.163 | .031 |
| **Weekend** | .598 | .3670 | 1.628 | .104 |
| **Weekday** | 0b | . | . | . |
| b. This coefficient is set to zero because it is redundant | | | | |

| **Table S3. PFP 3: Have to make sure your child did not eat too much food** | | | | |
| --- | --- | --- | --- | --- |
| Model Term | Coefficient | Std. Error | t | Sig. |
|  |  |  |  |  |
| **Intercept** | -3.274 | 1.9629 | -1.668 | .096 |
| **State stress** | .076 | .3113 | .243 | .808 |
| **State Negative Affect** | -.239 | .4673 | -.512 | .609 |
| **Reducing Conflict Goal =1** | .525 | .2948 | 1.781 | .075 |
| **Reducing Conflict Goal =0** | 0b | . | . | . |
| **Negative atmosphere** | -.133 | .3743 | -.357 | .721 |
| **Neutral atmosphere** | -.083 | .3881 | -.214 | .830 |
| **Positive atmosphere** | 0b | . | . | . |
| **Snack** | .972 | .2702 | 3.595 | <.001 |
| **Meal** | 0b | . | . | . |
| **Public setting** | -.127 | .7337 | -.173 | .863 |
| **Private setting** | 0b | . | . | . |
| **Probability of avid eating** | .561 | 1.2331 | .455 | .650 |
| **Parent age** | .000 | .0391 | .006 | .995 |
| **Child age** | -.002 | .0209 | -.075 | .940 |
| **Weekend** | .003 | .2784 | .012 | .991 |
| **Weekday** | 0b | . | . | . |
| b. This coefficient is set to zero because it is redundant | | | | |

| **Table S4. PFP 4: Offer your child a treat or reward for trying a new food** | | | | |
| --- | --- | --- | --- | --- |
| Model Term | Coefficient | Std. Error | t | Sig. |
|  |  |  |  |  |
| **Intercept** | -3.874 | 2.7570 | -1.405 | .160 |
| **State stress** | .340 | .4053 | .839 | .402 |
| **State Negative Affect** | .118 | .6070 | .195 | .845 |
| **Reducing Conflict Goal =1** | .383 | .4069 | .941 | .347 |
| **Reducing Conflict Goal =0** | 0b | . | . | . |
| **Negative atmosphere** | .489 | .4862 | 1.006 | .315 |
| **Neutral atmosphere** | -.976 | .6255 | -1.561 | .119 |
| **Positive atmosphere** | 0b | . | . | . |
| **Snack** | -2.050 | .5628 | -3.642 | <.001 |
| **Meal** | 0b | . | . | . |
| **Public setting** | .280 | .8369 | .335 | .738 |
| **Private setting** | 0b | . | . | . |
| **Probability of avid eating** | -.174 | 1.8490 | -.094 | .925 |
| **Parent age** | -.062 | .0540 | -1.155 | .248 |
| **Child age** | .055 | .0300 | 1.835 | .067 |
| **Weekend** | .952 | .3766 | 2.527 | .012 |
| **Weekday** | 0b | . | . | . |
| b. This coefficient is set to zero because it is redundant | | | | |

| **Table S5. PFP 5: Trick or bribe your child into eating more than they wanted to** | | | | |
| --- | --- | --- | --- | --- |
| Model Term | Coefficient | Std. Error | t | Sig. |
|  |  |  |  |  |
| **Intercept** | -4.054 | 2.3636 | -1.715 | .087 |
| **State stress** | .208 | .3758 | .553 | .580 |
| **State Negative Affect** | -.429 | .6075 | -.706 | .480 |
| **Reducing Conflict Goal =1** | .989 | .3705 | 2.670 | .008 |
| **Reducing Conflict Goal =0** | 0b | . | . | . |
| **Negative atmosphere** | .419 | .4535 | .923 | .356 |
| **Neutral atmosphere** | -.675 | .5537 | -1.220 | .223 |
| **Positive atmosphere** | 0b | . | . | . |
| **Snack** | -3.135 | .7792 | -4.023 | <.001 |
| **Meal** | 0b | . | . | . |
| **Public setting** | -.639 | .7584 | -.843 | .399 |
| **Private setting** | 0b | . | . | . |
| **Probability of avid eating** | 3.229 | 1.6272 | 1.984 | .048 |
| **Parent age** | -.037 | .0451 | -.829 | .407 |
| **Child age** | -.014 | .0251 | -.574 | .566 |
| **Weekend** | .779 | .3260 | 2.388 | .017 |
| **Weekday** | 0b | . | . | . |
| b. This coefficient is set to zero because it is redundant | | | | |

| **Table S6. PFP 6: Choose to prepare separate food that you knew your child would enjoy eating** | | | | |
| --- | --- | --- | --- | --- |
| Model Term | Coefficient | Std. Error | t | Sig. |
|  |  |  |  |  |
| **Intercept** | -3.569 | 1.7460 | -2.044 | .041 |
| **State stress** | .256 | .2638 | .970 | .332 |
| **State Negative Affect** | -.139 | .3835 | -.363 | .717 |
| **Reducing Conflict Goal =1** | .817 | .2566 | 3.186 | .001 |
| **Reducing Conflict Goal =0** | 0b | . | . | . |
| **Negative atmosphere** | -.318 | .3383 | -.941 | .347 |
| **Neutral atmosphere** | .026 | .3407 | .076 | .940 |
| **Positive atmosphere** | 0b | . | . | . |
| **Snack** | -.256 | .2511 | -1.019 | .308 |
| **Meal** | 0b | . | . | . |
| **Public setting** | -.017 | .7473 | -.023 | .982 |
| **Private setting** | 0b | . | . | . |
| **Probability of avid eating** | 1.136 | 1.1232 | 1.011 | .312 |
| **Parent age** | .022 | .0347 | .627 | .531 |
| **Child age** | -.005 | .0190 | -.276 | .783 |
| **Weekend** | .408 | .2269 | 1.797 | .073 |
| **Weekday** | 0b | . | . | . |
| b. This coefficient is set to zero because it is redundant | | | | |

| **Table S7. PFP7: Allow your child to choose a separate meal or different food because they did not want to eat what was offered.** | | | | |
| --- | --- | --- | --- | --- |
| Model Term | Coefficient | Std. Error | t | Sig. |
|  |  |  |  |  |
| **Intercept** | -2.820 | 2.2642 | -1.245 | .213 |
| **State stress** | .527 | .4102 | 1.285 | .199 |
| **State Negative Affect** | -.369 | .5692 | -.649 | .517 |
| **Reducing Conflict Goal =1** | 1.857 | .4185 | 4.437 | <.001 |
| **Reducing Conflict Goal =0** | 0b | . | . | . |
| **Negative atmosphere** | -.578 | .5464 | -1.057 | .291 |
| **Neutral atmosphere** | -.660 | .5901 | -1.118 | .264 |
| **Positive atmosphere** | 0b | . | . | . |
| **Snack** | -.940 | .4853 | -1.937 | .053 |
| **Meal** | 0b | . | . | . |
| **Public setting** | 2.111 | .6390 | 3.304 | <.001 |
| **Private setting** | 0b | . | . | . |
| **Probability of avid eating** | -1.164 | 1.4759 | -.789 | .431 |
| **Parent age** | -.031 | .0451 | -.689 | .491 |
| **Child age** | .017 | .0248 | .704 | .482 |
| **Weekend** | .873 | .3471 | 2.514 | .012 |
| **Weekday** | 0b | . | . | . |
| b. This coefficient is set to zero because it is redundant | | | | |

| **Table S8. PFP8: Give your child food in order to calm them down or help manage their behaviour.** | | | | |
| --- | --- | --- | --- | --- |
| Model Term | Coefficient | Std. Error | t | Sig. |
|  |  |  |  |  |
| **Intercept** | -4.514 | 2.5967 | -1.738 | .083 |
| **State stress** | .507 | .3863 | 1.312 | .190 |
| **State Negative Affect** | -.196 | .5619 | -.349 | .727 |
| **Reducing Conflict Goal =1** | 1.472 | .4125 | 3.567 | <.001 |
| **Reducing Conflict Goal =0** | 0b | . | . | . |
| **Negative atmosphere** | 1.063 | .4251 | 2.502 | .013 |
| **Neutral atmosphere** | -.104 | .5149 | -.202 | .840 |
| **Positive atmosphere** | 0b | . | . | . |
| **Snack** | 1.782 | .3620 | 4.922 | <.001 |
| **Meal** | 0b | . | . | . |
| **Public setting** | 1.120 | .7656 | 1.462 | .144 |
| **Private setting** | 0b | . | . | . |
| **Probability of avid eating** | 3.787 | 1.7583 | 2.154 | .032 |
| **Parent age** | -.064 | .0511 | -1.259 | .208 |
| **Child age** | -.023 | .0280 | -.816 | .415 |
| **Weekend** | .255 | .3495 | .729 | .466 |
| **Weekday** | 0b | . | . | . |
| b. This coefficient is set to zero because it is redundant | | | | |
